# Self-Relevance of Oral Health Among 12-Year-Old Schoolchildren in Nellore: A Cross-Sectional Study

**DOI:** 10.7759/cureus.81741

**Published:** 2025-04-05

**Authors:** Srinivasulu Gomasani, Shilpa Gollaprolu, Krishna Kumar R V S, Prasanth Punamalli, Lasya Suma P

**Affiliations:** 1 Public Health Dentistry, Narayana Dental College and Hospital, Nellore, IND; 2 Conservative Dentistry and Endodontics, Narayana Dental College and Hospital, Nellore, IND

**Keywords:** dental caries, oral health, oral hygiene, quality of life, school children

## Abstract

Introduction: The integration of health-related quality-of-life evaluations into clinical research has led to the development of disease-specific assessment tools. The Children Oral Health-Related Quality of Life framework comprises multidimensional scales that measure the negative impact of oral health issues on a child’s well-being and daily functioning. One such tool is the Child Perceptions Questionnaire for children aged 11-14 years (CPQ11-14).

Aim: This study aims to assess the association between oral health status and oral health-related quality of life (OHRQoL) among 12-year-old schoolchildren in the Nellore district of Andhra Pradesh.

Methods: A cross-sectional survey was conducted among 12-year-old schoolchildren attending government schools in the Nellore district, Andhra Pradesh. Child perception was evaluated using the Telugu-translated CPQ11-14 questionnaire. Oral health status was clinically examined through assessment of oral hygiene status and dental caries. Spearman correlation was conducted to assess the relationship between CPQ11-14 scores and oral health status.

Results: A total of 450 schoolchildren participated in the study. The overall mean CPQ11-14 score was 17.22. CPQ scores showed a weak positive correlation with the Simplified Oral Hygiene Index (OHI-S) (0.005) and the Decayed, Missing, and Filled Teeth Index (DMFT) (0.05).

Conclusion: Poor oral health status and dental caries had a negative impact on the OHRQoL of children.

## Introduction

Oral diseases in children can affect essential day-to-day functions, such as eating, chewing, swallowing, speaking, and smiling. Subsequently, they affect a child’s physical, emotional, social, and psychosocial well-being [1]. Oral health-related quality of life (OHRQoL) is an integral part of the overall health and well-being of individuals and should be considered a subjective component when assessing oral health status [2]. There are various measures of OHRQoL, with the Child Perceptions Questionnaire (CPQ) being the most commonly used, followed by the Child Oral Impacts on Daily Performances (C-OIDP) and the Child Oral Health Impact Profile (COHIP). The CPQ has been validated in multiple versions for different age groups, including CPQ8-10, CPQ11-14, CPQ14-16, and CPQ 28-100 [3]. The CPQ11-14 is a COHQoL questionnaire composed of 37 items that assess the repercussions of oral health problems on the quality of life of children between 11 and 14 years old [4].

A new short-form CPQ11-14 questionnaire consisting of 16 items (with each domain containing four items) has been developed to broaden its application. This version reduces the costs and time associated with printing and data collection, while also lowering the likelihood of unit or item nonresponse [5]. It has demonstrated excellent criterion validity and construct validity [6]. However, limited data are available on the impact of oral diseases on the quality of life among 12-year-old schoolchildren using the Telugu version of the CPQ11-14 questionnaire. Therefore, the present study aims to assess the association between oral health status and OHRQoL among 12-year-old schoolchildren in the Nellore district of Andhra Pradesh.

## Materials and methods

Study design

A cross-sectional survey was conducted over a one-month period, from July to August 2023, among schoolchildren aged 12 years attending government schools in the Nellore district of Andhra Pradesh.

Sample size estimation

The sample size for the study was calculated for a finite population of 43,000, using a 95% confidence level, 5% precision, and an estimated dental caries prevalence of 53.1% (as reported in the National Oral Health Survey and Fluoride Mapping, 2002-2004). The calculated sample size was 450.

Sampling procedure

The required sample was drawn from across the district using a stratified cluster random sampling procedure, with stratification based on basic demographic characteristics, namely, rural and urban public schools. The district comprises three administrative divisions: Gudur, Kavali, and Nellore. A total of five mandals from each administrative division were randomly included in the sampling frame. The list of schools for each selected mandal was obtained from the District Educational Office of Nellore. One school from each mandal was randomly selected to serve as a sampling unit. The sample of 450 children was equally distributed among the 15 schools from the 15 mandals selected across the three administrative divisions of the Nellore district. The primary investigator personally selected the participants, choosing every third student until the estimated sample size was achieved (i.e., 30 students from each school).

Inclusion criteria

Schoolchildren aged 12 years with fully erupted permanent teeth were included in the study.

Exclusion criteria

Schoolchildren who did not consent to participate or were absent during the study period were excluded.

Data collection

Data were recorded using a specially designed proforma consisting of four parts: (1) sociodemographic data, including assessment of socioeconomic status (SES) using the Kuppuswamy scale [7]; (2) global rating of oral health using the short form of the CPQ11-14 [6], which includes four subdomains (oral symptoms (OS), functional limitation (FL), emotional well-being (EWB), and social well-being (SWB)), each rated on a five-point Likert scale (0 = never, 1 = once/twice, 2 = sometimes, 3 = often, and 4 = every day/almost every day); (3) clinical examination to assess oral hygiene status using the Simplified Oral Hygiene Index (OHI-S), categorized as poor, fair, and good [8]; and (4) clinical examination for dental caries using the Decayed, Missing, and Filled Teeth Index (DMFT; WHO modification 1987).A DMFT score of 0 indicates no caries, while a score of 1 or more indicates caries presence [9].

Adaptation and translation of the short-form Child Perceptions Questionnaire (CPQ11-14)

To measure the OHRQoL of children in the Nellore district of Andhra Pradesh, the questionnaires were translated into Telugu. Following standard guidelines, two bilingual translators with experience in health-related questionnaire translation independently translated the instrument (one Telugu speaker fluent in English and one English speaker fluent in Telugu). A panel of specialists then reviewed and synthesized the translations to ensure conceptual and item equivalence, with particular attention to word meanings in Telugu to ensure cultural appropriateness and identify potential comprehension difficulties. To assess the equivalence between the original and back-translated versions, a Telugu translator fluent in English conducted a third assessment. Operational equivalence was evaluated using a sample of 45 children aged 11-14 years who were not part of the main study sample. The Telugu versions of the CPQ11-14 short form demonstrated satisfactory conceptual and semantic equivalence with the original, confirming its reliability and validity for use among Telugu-speaking children.

Test-retest reliability

The test-retest reliability of the CPQ11-14 was assessed by re-administering the questionnaire after three days to 5% of the sample. The Cronbach’s alpha coefficient was 0.89, indicating excellent internal consistency, comparable to the values reported in the original Canadian study [10].

Training and calibration

Before the start of the study, the examiner underwent training and calibration for the indices used. The intra-class correlation coefficient (ICC) was 0.86 for the OHI-S and 0.92 for DMFT. During the survey, data collection was repeated on 5% of the sample to assess intra-examiner reproducibility, with a minimum interval of two days between examinations. The resulting ICC was 0.96.

Examination procedure

A type III clinical examination, as recommended by the American Dental Association (ADA), was used. This method involves inspection using a mouth mirror, explorer, and adequate illumination and is the most commonly employed technique in public health surveys. All examinations were conducted by the primary investigator, with data recorded by an assistant.

Statistical analysis

IBM SPSS Statistics for Windows, Version 22.0 (Released 2013; IBM Corp., Armonk, NY, United States) was used for data analysis. Descriptive analyses were initially performed to assess the prevalence of oral impacts and to calculate measures of central tendency (means and standard deviations) for both total and individual domain scores of the Telugu CPQ11-14. Spearman rank-order correlation was then conducted to assess the relationship between oral diseases and both the overall mean CPQ11-14 score and individual domain scores.

## Results

A total of 450 schoolchildren, aged 12 years, participated in the study. The majority of participants were girls (72.2%), and most belonged to the lower middle socioeconomic class (62.9%) (Table 1).

**Table 1:** Sample distribution according to sociodemographic details.

Variable	n	%
Gender		
Male	125	27.8
Female	325	72.2
Socioeconomic status		
Upper	0	0
Upper middle	33	7.3
Lower middle	283	62.9
Upper lower	130	28.9
Lower	4	0.9

The mean OHI-S score was 1.23 ± 0.71. The majority of participants (44%) had fair oral hygiene status. The mean DMFT score was 0.51 ± 0.50, with 51.4% of the children having one or more carious lesions (Table 2). Regarding OHRQoL, based on the CPQ11-14 index, higher scores indicate worse OHRQoL.

**Table 2:** Distribution of the study population according to the clinical parameters. DMFT: Decayed, Missing, and Filled Teeth Index.

Clinical indices	n	%	Mean ± SD
				Oral hygiene status			1.23 ± 0.71
Poor (3.1-6.0)	74	16.4					
Fair (1.3-3.0)	198	44					
Good (0.0-1.2)	178	39.6					
DMFT			0.51 ± 0.50
Caries-free (0 cavity)	219	48.6	
With caries (1 or more)	231	51.4	

The highest overall mean CPQ11-14 score for SES was observed in schoolchildren from the lower class (24.25). When domain-specific CPQ scores were considered, the EWB component had the highest impact (6.25). Schoolchildren with poor oral hygiene status (19.76) and those with caries (17.67) showed higher mean CPQ11-14 scores. For schoolchildren with poor oral hygiene, the highest impact was seen in the EWB component (5.07), followed by OS (4.55), and the lowest impact was in SWB (2.90). In schoolchildren with caries, the EWB component again had the highest impact (4.85), followed by OS (3.76), and the least impact was in SWB (2.61) (Table 3).

**Table 3:** Overall and domain-specific mean CPQ11-14 scores according to socioeconomic status and clinical parameters. CPQ: Child Perceptions Questionnaire, OS: oral symptoms, FL: functional limitation, EWB: emotional well-being, SWB: social well-being, OHI-S: Simplified Oral Hygiene Index, DMFT: Decayed, Missing, and Filled Teeth Index.

Socioeconomic status and clinical parameters	CPQ11-14	OS	FL	EWB	SWB
	Mean (95% CI)	Mean (95% CI)	Mean (95% CI)	Mean (95% CI)	Mean (95% CI)
Socioeconomic status					
Upper	0	0	0	0	0
Upper middle	16.74 (15.20-18.29)	3.56 (3.10-4.04)	2.90 (2.50-3.34)	4.34 (3830-4.84)	2.65 (2.24-3.07)
Lower middle	17.28 (16.15-18.35)	3.68 (3.35-3.39)	3.39 (3.05-3.72)	4.70 (4.39-5.04)	2.43 (2.16-2.71)
Upper lower	17.67 (14.70-20.50)	3.91 (2.89-4.79)	2.97 (2.11-3.82)	4.79 (3.76-5.71)	2.61 (1.89-3.33)
Lower	24.25 (12.00-36.00)	5.00 (2.00-8.00)	4.50 (1.00-8.00)	6.25 (2.00-11.00)	5.50 (2.00-10.00)
OHI-S					
Poor	19.76 (17.04-22.36)	4.55 (3.81-5.23)	3.87 (3.13-4.57)	5.07 (4.19-5.78)	2.90 (2.24-3.51)
Fair	16.74 (15.39-17.97)	3.46 (3.08-3.85)	3.09 (2.70-3.58)	4.52 (4.10-4.90)	2.48 (2.11-2.84)
Good	16.72 (14.21-19.29)	3.54 (3.11-3.94)	3.16 (2.77-3.55)	4.56 (4.18-4.95)	2.46 (2.15-2.78)
DMFT					
Caries-free	16.80 (14.37-19.11)	3.61 (3.07-4.13)	3.18 (2.64-3.70)	4.38 (3.80-4.88)	2.47 (1.98-2.98)
With caries	17.67 (15.36-20.04)	3.76 (3.25-4.29)	3.26 (2.76-3.77)	4.85 (4.31-5.41)	2.61 (2.14-3.11)

The correlation between the overall CPQ11-14 score and domain-specific scores with clinical parameters showed a weak positive correlation of OHI-S (0.005) and DMFT (0.053), neither of which were statistically significant. However, when the domain-specific scores were correlated with clinical parameters, children with caries (DMFT) showed a positive correlation with the EWB (0.103) component of CPQ, which was statistically significant (Table 4). 

**Table 4:** Correlation of overall CPQ11-14 score and domain-specific scores with clinical parameters using Spearman correlation. OHI-S: Simplified Oral Hygiene Index, DMFT: Decayed, Missing, and Filled Teeth Index.

Clinical Indices	Total scale (16 items)	OS (4 items)	FL (4 items)	EWB (4 items)	SWB (4 items)
	r (95% CI)	r (95% CI)	r (95% CI)	r (95% CI)	r (95% CI)
OHI-S	0.005 (-0.184 to 0.021)	0.006 (-0.180 to 0.013)	0.042 (-0.156 to 0.061)	0.039 (-0.149 to 0.048)	0.079 (-0.127 to 0.088)
DMFT	0.053 (-0.036 to 0.142)	0.025(-0.068 to 0.107)	0.015 (-0.084 to 0.108)	0.103 (-0.006 to 0.195)*	0.019 (-0.075 to 0.103)

## Discussion

The present study aims to assess the association between oral health status and OHRQoL among 12-year-old schoolchildren in both urban and rural areas of the Nellore district, Andhra Pradesh, India, using the short form of the CPQ11-14. In India, comprehensive information regarding oral health is limited, as regular data collection is not conducted. The only national oral health survey was carried out in 2002, and no subsequent national-level surveys have been conducted in India or Andhra Pradesh [11].

This survey focuses on one of the WHO index age groups (12 years), where the oral disease pattern is expected to reflect evolving living conditions and the adoption of modern lifestyles. This study concentrates on two of the most prevalent chronic oral diseases in this age group: dental caries and gingival issues.

Oral health impacts are particularly relevant for the specific age group under investigation (12 years), considering their developmental stage. Developmental changes inevitably affect HRQoL between childhood and adolescence [12]. As children mature and age, they develop a more nuanced understanding and perception of health and illness [13]. Consequently, their views on health and quality of life evolve [12,14], potentially making younger children more sensitive to oral symptoms compared to older age groups.

The study was conducted in 15 urban and rural areas of Nellore district, which represent diverse sociocultural and economic conditions. The data collected indicated that most children in the study were from the lower-middle class. The analysis of CPQ scores in relation to SES showed that children from the lower class had poorer OHRQoL. Parental education and income may be factors influencing health and healthcare service utilization, which in turn could contribute to poor oral health and lower OHRQoL. Locker et al. [15] observed that low parental socioeconomic position was significantly associated with greater dental caries and periodontal disease experience. Furthermore, previous studies have reported that individuals from low-income households have poorer general and oral health than those from high-income households [16],which aligns with the findings of our study.

Gum problems were significant oral conditions affecting children's OHRQoL. More than one-fifth of children reported that gum disease and bleeding had an impact on their lives, especially in relation to cleaning, which is a challenge faced by more than half of all children. Children who struggle to brush their teeth due to gum inflammation are unlikely to develop proper oral hygiene, as brushing may cause bleeding, leading to the persistence or worsening of their gum issues [17]. This problem cannot be addressed by traditional dental treatment alone without understanding the behavioral effects of oral health impacts. Interestingly, impacts related to social dimensions, such as disruptions in studies and social interactions, were less common and less severe. Schor suggested that children's social performance depends more on their physical and psychological well-being than on their social interactions, in contrast to adults [18].

Another interesting finding in our study was that, when analyzing the impact of caries on OHRQoL, children with dental caries experienced a higher impact, despite the prevalence of caries being low compared to the national oral health survey conducted in 2002 in Andhra Pradesh, India. This finding contrasts with studies by Tadakamadla et al. [1], Jain et al. [2], and Kassis et al. [19],where dental caries experience was not found to be related to OHRQoL. Direct comparison with previous studies was challenging due to the use of different indices for assessing and analyzing caries status, as well as the use of the short-form CPQ11-14 in the current study. Jokovic et al. [6] validated the original full-length version and found a significant correlation between the number of decayed tooth surfaces and overall scale scores (r = 0.64; p = 0.007). In Brazil, Goursand et al. [4] demonstrated that the long-form CPQ11-14 could distinguish between children with and without untreated dental caries, with those having untreated caries showing higher average total scores compared to those without untreated caries (p < 0.05).

In our study, we found a significant correlation between dental caries and the EWB subscale, but no significant correlation with the other domains. This contrasts with the study by Brown and Al-Khayal [20], which found a significant correlation only between the DMFT and the OS subscales, but not with other domains (FL, EWB, and SWB) in Arabic children. Clinical indicators measure disease, while OHRQoL indicators concentrate on health and well-being [21,22].Consequently, although periodontal disease and dental caries are quite common, they may not significantly hinder a child's ability to perform daily activities in the early stages. This suggests that the relationship between OHRQoL and clinical indicators should be interpreted with caution. The inconsistencies observed between clinical data and OHRQoL may not stem from the psychometric properties of the measures, but rather from the influence of other factors, such as personal, social, and environmental variables [23].

Limitations

The SES of the study participants was assessed, and the majority of the participants belonged to the lower and lower-middle classes. Additionally, the study was conducted specifically in government schools.

## Conclusions

The present study concluded that schoolchildren from lower-middle class, those with poor oral hygiene status, and those with dental caries had poorer OHRQoL. These findings suggest that oral health interventions and education are essential for improving both dental health and overall quality of life in children. Strengthening preventive measures and fostering good oral hygiene practices from an early age could help mitigate these negative impacts. Further studies with larger sample sizes could provide more detailed insights into these associations, thereby guiding more effective public health strategies.

## Appendices

Short-form CPQ11-14

Questions About Oral Problems

In the past three months, how often have you had:

1. Pain in your teeth, lips, jaws, or mouth?

( ) Never

( ) Once or twice

( ) Sometimes

( ) Often

( ) Every or almost every day

2. Sores/ulcers in your mouth?

( ) Never

( ) Once or twice

( ) Sometimes

( ) Often

( ) Every or almost every day

3. Bad breath?

( ) Never

( ) Once or twice

( ) Sometimes

( ) Often

( ) Every or almost every day

4. Food stuck in or between your teeth?

( ) Never

( ) Once or twice

( ) Sometimes

( ) Often

( ) Every or almost every day

Has this happened because of your teeth, lips, jaws, or mouth?

In the past three months, how often have you:

5. Taken longer than others to eat a meal?

( ) Never

( ) Once or twice

( ) Sometimes

( ) Often

( ) Every or almost every day

In the past three months, because of your teeth, lips, mouth, or jaws, is it:

6. Difficult to bite or chew food like apples and corn?

( ) Never

( ) Once or twice

( ) Sometimes

( ) Often

( ) Every or almost every day

7. Difficult to say any words?

( ) Never

( ) Once or twice

( ) Sometimes

( ) Often

( ) Every or almost every day

8. Difficult to drink or eat hot or cold foods?

( ) Never

( ) Once or twice

( ) Sometimes

( ) Often

( ) Every or almost every day

Questions About Feelings

Have you had the feeling because of your teeth, lips, jaws, or mouth?

In the past three months, how often have you:

9. Felt irritable or frustrated?

( ) Never

( ) Once or twice

( ) Sometimes

( ) Often

( ) Every or almost every day

10. Felt shy?

( ) Never

( ) Once or twice

( ) Sometimes

( ) Often

( ) Every or almost every day

11. Been upset?

( ) Never

( ) Once or twice

( ) Sometimes

( ) Often

( ) Every or almost every day

12. Have you been concerned what other people think about your teeth, lips, mouth, or jaws?

( ) Never

( ) Once or twice

( ) Sometimes

( ) Often

( ) Every or almost every day

Questions About Your Spare Time Activities and Being With Other People

Have you had these experiences because of your teeth, lips, jaws, or mouth?

13. Avoid smiling or laughing when around other children?

( ) Never

( ) Once or twice

( ) Sometimes

( ) Often

( ) Every or almost every day

14. Argued with other children or your family?

( ) Never

( ) Once or twice

( ) Sometimes

( ) Often

( ) Every or almost every day

15. Other children teased or called names because of your teeth, lips, jaws, or mouth?

( ) Never

( ) Once or twice

( ) Sometimes

( ) Often

( ) Every or almost every day

16. Other children asked you questions about your teeth, lips, jaws, or mouth?

( ) Never

( ) Once or twice

( ) Sometimes

( ) Often

( ) Every or almost every day
